# Chemokine Expression in Well‐Differentiated Liposarcoma May Be Involved in the Tumorigenesis of Lymphoplasmacytic Lymphoma: A Case Study

**DOI:** 10.1002/cnr2.70129

**Published:** 2025-02-02

**Authors:** Kota Washimi, Rika Kasajima, Shinya Sato, Yutaka Nezu, Hiroyuki Takahashi, Rika Sakai, Naoya Nakamura, Masayuki Takagi, Chie Hasegawa, Emi Yoshioka, Yoichiro Okubo, Kotoe Katayama, Seiya Imoto, Tomoyuki Yokose, Yohei Miyagi

**Affiliations:** ^1^ Department of Pathology Kanagawa Cancer Center Yokohama Japan; ^2^ Molecular Pathology and Genetics Division Kanagawa Cancer Center Research Institute Yokohama Japan; ^3^ Division of Health Medical Intelligence, Human Genome Center Institute of Medical Science, The University of Tokyo Tokyo Japan; ^4^ Division of Advanced Cancer Therapeutics Kanagawa Cancer Center Research Institute Yokohama Japan; ^5^ Department of Musculoskeletal Tumor Surgery Yokohama City University Yokohama Japan; ^6^ Department of Hematology and Medical Oncology Kanagawa Cancer Center Yokohama Japan; ^7^ Department of Pathology Tokai University School of Medicine Isehara Japan; ^8^ Department of Pathology National Hospital Organization Shizuoka Medical Center Shizuoka Japan; ^9^ Laboratory of Sequence Analysis, Human Genome Center Institute of Medical Science, The University of Tokyo Tokyo Japan; ^10^ Department of Pathology Odawara Municipal Hospital Odawara Japan

**Keywords:** chemokine, lymphoplasmacytic lymphoma, MDM2, well‐differentiated liposarcoma

## Abstract

**Background:**

Liposarcoma and lymphoma are very rare tumors, and their combination is extremely rare. Moreover, there have been no reports of liposarcoma and lymphoma occurring in the same region.

**Case:**

A 58‐year‐old man presented to Kanagawa Cancer Center with a mass in his left thigh and underwent a needle biopsy. Histological analysis showed an increase in the number of small lymphocytes and plasma cells; immunohistochemical analysis showed an increase in CD20‐positive cells with Lambda light‐chain restriction; therefore, the diagnosis of B‐cell malignancy with plasma cell differentiation was made. A bone marrow biopsy specimen showed infiltration of atypical cells of the same phenotype and increased serum IgM‐M levels; therefore, a diagnosis of Waldenström macroglobulinemia/lymphoplasmacytic lymphoma (LPL) was made. The needle biopsy specimen showed scattered CDK4‐positive cells in the background of the lymphoma cells and sporadic *MDM2* signal amplification on fluorescence in situ hybridization, suggesting mixed well‐differentiated liposarcoma (WDL). Tumor resection was performed. The tumor contained a mixture of WDL and LPL areas. RNA sequencing revealed upregulated expression of chemokine genes, including *CCL5*, *CCL18*, and *CCL19*, in WDL and that of the corresponding chemokine receptor genes *CCR4*, *CCR6*, and *CCR7* in the lymphoma cells.

**Conclusion:**

Chemokine–chemokine receptor axes may be involved in the pathogenesis of LPL cell‐infiltrating WDL. This is an extremely rare case, and we have reported some considerations regarding the tumorigenesis of LPL cell‐infiltrating WDL.

## 1 Introduction

Well‐differentiated liposarcomas (WDLs) are adipocytic tumors with locally progressive growth. Identification of hyperchromatic stromal cells is important for their diagnosis. *MDM2* amplification identified via fluorescence in situ hybridization (FISH) is useful to differentiate WDLs from benign lipomas [1, 2]. WDL is a rare tumor with an incidence of 0.31–0.35 per 100 000 per year [3]. Inflammatory WDL is a rare subtype of WDL with prominent lymphocytic and plasma cell infiltration of the retroperitoneum and paratestis [1, 4]. Lymphoplasmacytic lymphoma (LPL) is a neoplasm comprising small B lymphocytes, plasmacytoid lymphocytes, and plasma cells, involving the bone marrow, lymph nodes, and spleen. The incidence of LPL is 3–7 per million per year [5]. Each tumor has a completely different origin, and no reports point to a relationship in terms of development. Herein, we describe a case in which well‐differentiated liposarcoma and low‐grade B‐cell lymphoma occurred in the same region. We report the relationship between the two tumors, including the results of gene mutation analysis.

## 2 Case

A 58‐year‐old man who tested positive for hepatitis B surface antigen (HBsAg) noticed a mass on his left thigh. A local physician suspected it to be an adipose tumor and recommended a contrast‐enhanced magnetic resonance imaging scan, which revealed a contrast effect. Subsequently, the patient was referred to the Kanagawa Cancer Center in Japan in 2022 for further examination and treatment. Molecular characterization of this case was conducted at the department of pathology and the research institute of Kanagawa Cancer Center from December 2022 to November 2024. This study received approval from the Research Ethics Committee (approval number: 2022 epidemiology‐101).

**FIGURE 1:**
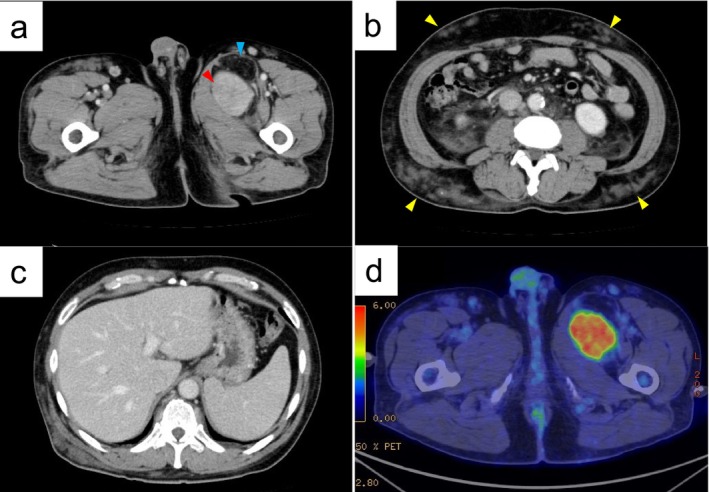
Contrast computed tomography (CT) findings. Borderline well‐defined mass lesion within the left thigh adductor muscle, with a 150‐mm large fatty dense area (blue arrow) and a lightly contrasted solid area (red arrow) (a). Multiple areas of soft density with indistinct boundaries within the subcutaneous fat of the trunk (yellow arrow) (b). Hepatosplenomegaly (c). Fluorodeoxyglucose positron emission tomography‐CT (PET‐CT) showing an accumulation with a maximum standard uptake value (SUVmax) of 6.84 in the solid area of the mass (d).

Contrast‐enhanced computed tomography (CT) revealed a 150‐mm large mass lesion with a mixture of fatty and 80‐mm large solid areas within the left thigh adductor muscle (Figure 1a). Multiple masses showed soft tissue density with indistinct boundaries within the subcutaneous adipose tissue of the trunk and temple regions (Figure 1b). Other findings included bilateral cervical, axillary, mediastinal, abdominal, para‐aortic to pelvic, and inguinal lymph node enlargement, as well as hepatosplenomegaly (Figure 1c). Fluorodeoxyglucose positron emission tomography‐CT (PET‐CT) showed an accumulation image with a maximum standard uptake value (SUVmax) of 6.84 in the solid area of the mass lesion within the left thigh adductor muscle (Figure 1d). Most of the swelling in the bilateral neck, axillary, mediastinal, abdominal para‐aortic to pelvic, and inguinal lymph nodes showed an accumulation image with an SUVmax of 2.80. An SUVmax of 1.6 was observed in areas of soft tissue density within the subcutaneous fatty tissue of the trunk and temple regions.

The CT results indicated the presence of dedifferentiated liposarcoma. A needle biopsy was performed on a solid area of the left thigh mass. It provided histopathological evidence of diffuse proliferation of small lymphocytes. Some intervening adipocytes and focal plasma cell proliferation were observed. Immunohistochemical analysis showed that the infiltrating lymphocytes were predominantly CD20‐positive and negative for CD10, Bcl‐6, cyclin D1, and Epstein–Barr virus‐encoded small RNA in in situ hybridization. The Ki‐67 index was approximately 10%–30%. Light chain restriction was observed, as evidenced by the presence of significantly larger numbers of Lambda‐positive cells compared to Kappa‐positive cells. Scattered small CD3‐ and CD5‐positive cells were observed in the background. Overall, the pathological diagnosis based on the needle biopsy was low‐grade B‐cell lymphoma. As an adipose tumor was suspected on imaging, CDK4 staining revealed a small number of CDK4‐positive cells intercalated within lymphocytes. Combined with imaging findings, FISH of *MDM2* revealed a small number of cells with amplified *MDM2* signals in the lymphoid aggregates, suggesting WDL.

A needle biopsy specimen suggested lymphoma; therefore, a bone marrow biopsy was performed. A cluster of small lymphocytes was observed in a portion of the periosteum, and the cells were positive for CD20 and CD79a, with a substantially greater number of Lambda‐positive cells than Kappa‐positive cells. The Ki‐67 index was low in the CD20‐positive cell area. The findings suggested bone marrow infiltration by lymphoma cells. The results of blood tests are presented in Table 1 and were as follows: IgG, 518 mg/dL (standard value: 861–1747 mg/dL); IgA, 44 mg/dL (93–393 mg/dL); IgM, 4664 mg/dL (36–245 mg/dL); free L chain (free Kappa, 8.2 mg/L (3.3–19.4 mg/L); free Lambda, 976 mg/L (5.7–26.3 mg/L); and Kappa/Lambda, 0.01 (0.26–1.65)); sIL2R, 996 U/mL (145–519 U/mL); AST, 11 U/L (13–30 U/L); ALT, 8 U/L (8–36 U/L); T‐bil, 0.6 mg/dL (0.4–1.5 mg/dL); TP, 9.2 g/dL (6.6–8.1 g/dL); and ALB, 3.4 g/dL (4.1–5.1 g/dL) [6, 7]. The IgM and sIL2R levels were high, and the free Lambda level was higher than the free Kappa level. We diagnosed LPL based on the presence of a B‐cell tumor with plasma cell differentiation, an infiltrate of atypical cells of the same phenotype in the bone marrow, and an increased serum IgM level.

**TABLE 1:** Blood test findings and standard values.

		Standard value
IgG	518 mg/dL	861–1747
IgA	44 mg/dL	93–393
IgM	4664 mg/dL	36–245
Free L chain		
Free Kappa	8.2 mg/L	3.3–19.4
Free Lambda	976 mg/L	5.7–26.3
Kappa/Lambda	0.01	0.26–1.65
sIL2R	996 U/mL	204–587
AST	11 U/L	13–30
ALT	8 U/L	8–36
T‐bil	0.6 mg/dL	0.4–1.5
TP	9.2 g/dL	6.6–8.1
ALB	3.4 g/dL	4.1–5.1

**FIGURE 2:**
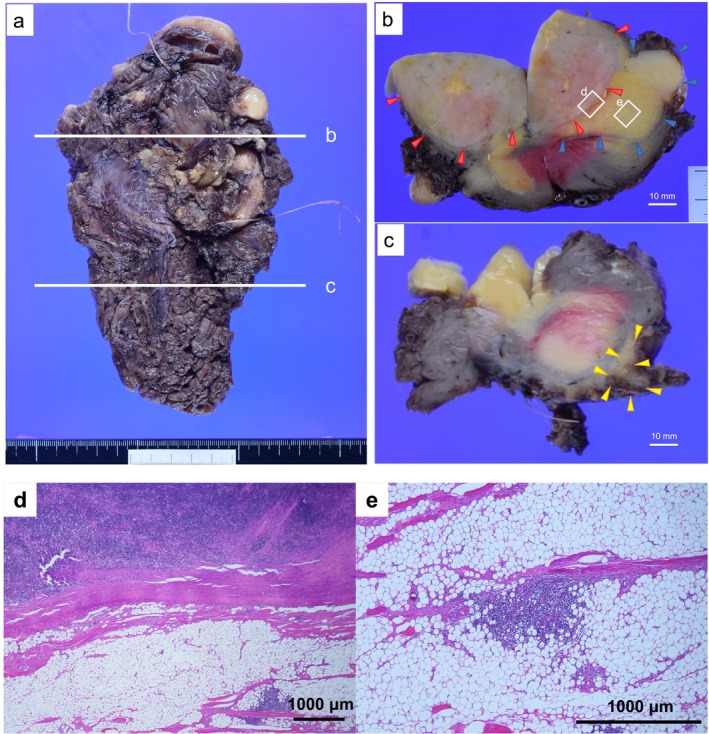
Specimen removed from the soft tissue tumor within the left thigh adductor muscle (a). Axial section cutout face of the tumor's superior side. Yellowish fatty areas (blue arrow) and grayish‐white solid areas (red arrow) with relatively well‐defined boundaries (b). A small amount of seemingly non‐neoplastic fatty tissue (yellow arrow) around the fatty tumor (c). Microscopic image of the area marked as “d” in (b) (bordered by a white frame). Low‐magnification image. A fibrous capsule formed between the fatty area with prominent mature adipocyte proliferation (lower part) and the grayish‐white solid area with prominent lymphocyte proliferation (upper part) (d). Microscopic image of the area marked as “e” in (b) (bordered by a white frame). Medium‐magnification image. Lymphocyte conglomerations scattered in islands around microvessels within the fatty area (e).

The case was considered a combination of WDL and LPL, and tumor resection was performed. The resected specimen showed a fatty tumor measuring 190 × 95 × 60 mm within the muscle and a grayish‐white‐toned solid area measuring 80 × 75 × 45 mm on the superior aspect of the mass (Figure 2a). The border between the fatty and solid areas was relatively clear; the fatty areas showed lobulated growth within the muscle tissue (Figure 2b). A small amount of gross non‐neoplastic fatty tissue was observed around the specimens (Figure 2c).

**FIGURE 3:**
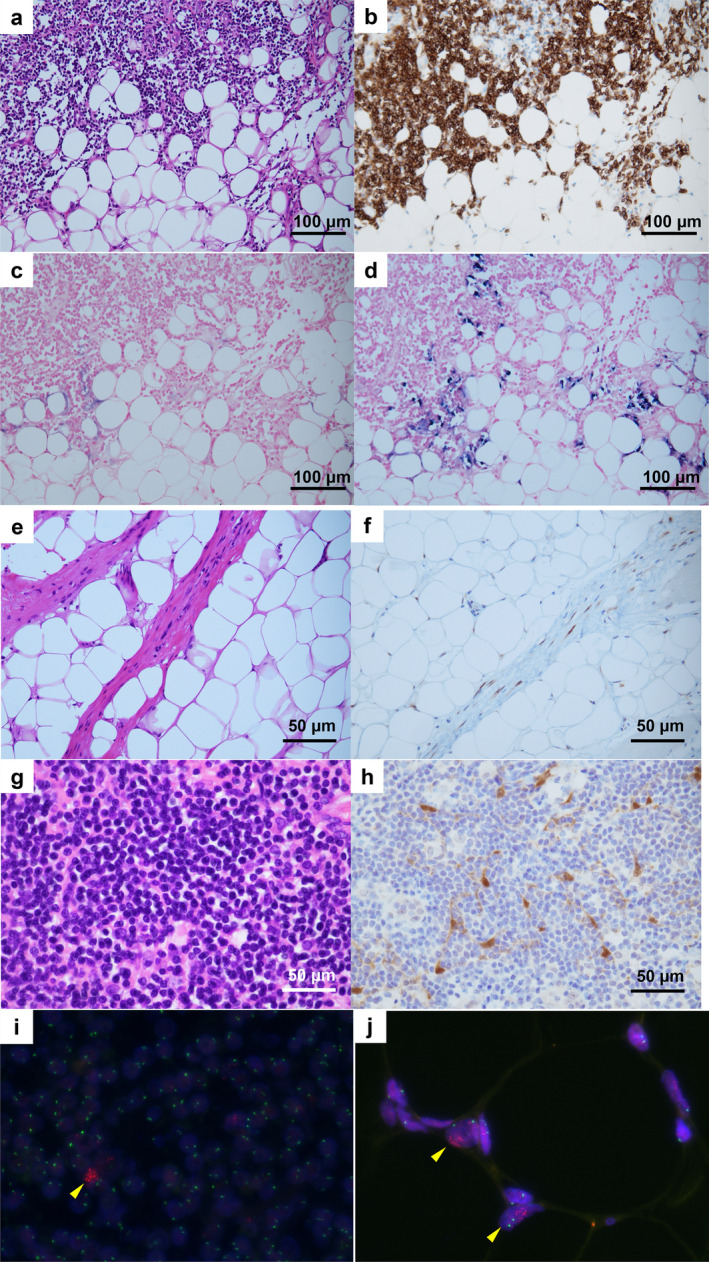
Predominance of small lymphocytes, with some intervening plasma cells, in histological section of the specimen. Original magnification at image acquisition: ×100 (a). Abundance of CD20‐positive cells. Original magnification at image acquisition: ×100 (b). Kappa staining showing a small number of positive cells. Original magnification at image acquisition: ×100 (c). Lambda‐positive cells were significantly more abundant than Kappa‐positive cells. Original magnification at image acquisition: ×100 (d). Fibrous septa were seen in the fatty areas, and hyperchromatic stromal cells were scattered. Original magnification at image acquisition: ×200 (e). Hyperchromatic stromal cells were partially positive for CDK4. Original magnification at image acquisition: ×200 (f). Small lymphocytes diffusely proliferated in the grayish‐white solid area. Original magnification at image acquisition: ×200 (g). A few CDK4‐positive cells were intercalated within small lymphocytes. Original magnification at image acquisition: ×200 (h). A few *MDM2* signal‐amplified cells (yellow arrow) were intercalated in lymphocytes, as determined via the FISH assay (i). Mature adipocytes in the fatty area showed amplification of *MDM2* (yellow arrow), as determined via the fluorescence in situ hybridization assay (j).

The fatty areas predominantly contained mature adipocytes, whereas the grayish‐white solid areas contained lymphocytes. A fibrous capsule was noted at the border (Figure 2d). Scattered clusters of small lymphocytes were observed in the form of islands around small blood vessels within the adipose tumor area (Figure 2e). A predominance of CD20‐positive cells and a high number of Lambda‐positive cells compared with that of Kappa‐positive cells was seen, suggesting lymphoma infiltration (Figure 3a–d). The adipose tumor area had relatively thick fibrous septa and scattered hyperchromatic stromal cells (Figure 3e). Immunohistochemistry revealed that the hyperchromatic stromal cells were positive for CDK4 (Figure 3f). The solid area at the superior aspect of the tumor was diffusely populated with small lymphocytes and was considered LPL because of similar histological findings and immunostaining results as those for the needle biopsy (Figure 3g). Surgical specimens also showed CDK4‐positive cells intervening in the area with diffuse proliferation of lymphocytes, and FISH revealed few intervening cells with amplified *MDM2* signals (Figure 3h,i). FISH of mature adipocytes in the adipose tumor area confirmed *MDM2* amplification, and this area was determined to be a WDL (Figure 3j).

**FIGURE 4:**
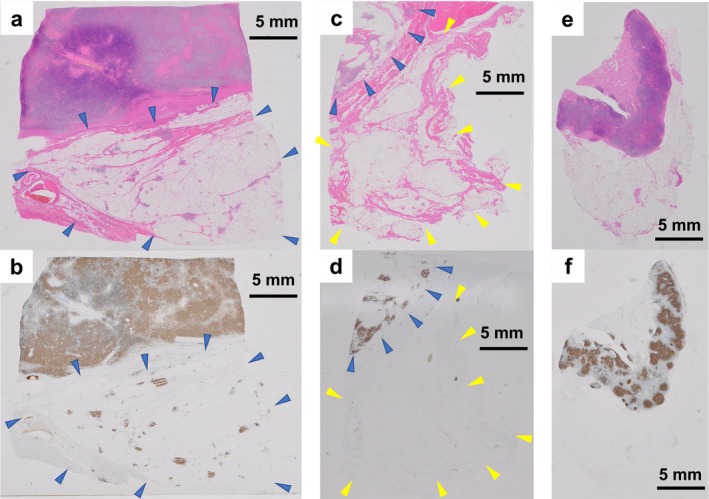
Findings of hematoxylin and eosin (HE) staining at the border between fatty and solid areas (a). CD20‐stained section of the specimen a. In the fatty tumor area (blue arrow), CD20‐positive lymphoma islands are visible (b). Findings of HE staining at the border between fatty areas and non‐neoplastic fatty tissue (c). CD20 staining of a section of the specimen (c). The fatty tumor area (blue arrow) shows foci of CD20‐positive lymphoma cells, whereas the surrounding non‐neoplastic fatty tissue (yellow arrow) shows no foci of CD20‐positive lymphoma cells (d). Findings of HE staining of dissected inguinal lymph nodes and surrounding non‐neoplastic fatty tissue (e). Nodular proliferation of CD20‐positive cells within the lymph nodes, with no infiltration of CD20‐positive cells in the surrounding fatty tissue (f).

The lymphoma cell infiltrates were scattered in islands within the WDL area (Figure 4a,b). However, no lymphoma infiltration was observed in the non‐neoplastic fatty tissue surrounding the tumor (Figure 4c,d). The left inguinal lymph node, which was dissected separately from the femoral tumor, presented nodular proliferation of CD20‐positive small lymphocytes. It was infiltrated by LPL cells as evident from light‐chain restriction, which was confirmed via Kappa and Lambda staining. Lymphoma cells did not infiltrate the non‐neoplastic fatty tissue surrounding the lymph nodes (Figure 4e,f).

**FIGURE 5:**
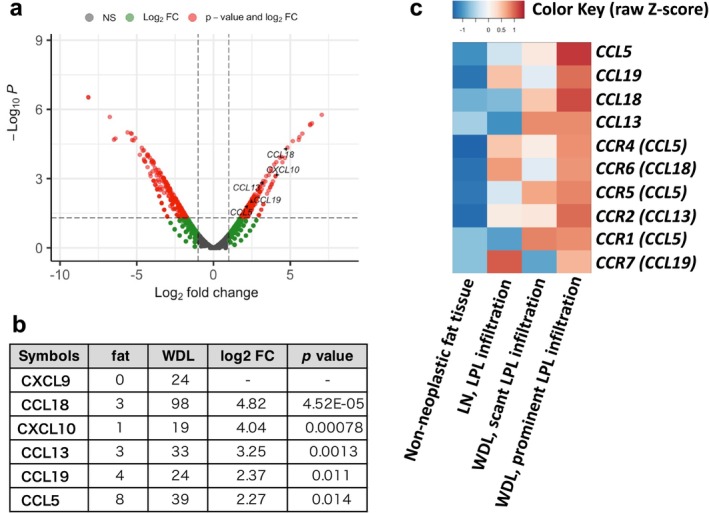
Transcriptome analyses using RNA sequencing. Differentially expressed genes (DEGs) between non‐neoplastic fat tissue and well‐differentiated liposarcoma (WDL) with scant lymphoma cell infiltration are shown in the volcano plot (a). DEGs with |log2 fold change (FC)| ≥ 1 and *p*‐value ≤ 0.05 are indicated with small red circles. Expression of genes with positive log2FC was upregulated in WDL. Expression of chemokine genes upregulated in WDL, demonstrated in terms of fragments per kilobase of exon per million reads mapped (FPKM) with log2FC and *p*‐value (b). A heatmap of comparative gene expression among four examined tissues, shown at the bottom of the map. Gene symbols are provided on the right side of the map, with corresponding chemokine ligand genes within parentheses for receptor genes (c).

No lymphoma cell infiltration was detected in the non‐neoplastic fatty tissue within the submitted surgical specimen, whereas infiltration was conspicuous in the WDL area. Therefore, RNA sequencing was performed using RNA extracted from four representative areas of formalin‐fixed paraffin‐embedded (FFPE) specimens: WDL with prominent lymphoma cell infiltration, WDL with scant lymphoma cell infiltration, lymph nodes with prominent lymphoma cell infiltration, and non‐neoplastic fat tissue. A comparison of gene expression was made between non‐neoplastic fat tissue and WDL with scant lymphoma cell infiltration, which was taken to represent WDL gene expression. This comparison revealed 13 754 differentially expressed genes (DEGs) with the absolute value of fragments per kilobase of exon per million reads mapped (FPKM) log2 fold change ≥ 1 and *p*‐value ≤ 0.05 (Figure 5a). The DEGs included multiple chemokine genes showing significantly higher expression in WDL than in non‐neoplastic fat tissue, including C‐C motif ligand (*CCL*)*5*, *CCL13*, *CCL18*, *CCL19*, C‐X‐C motif ligand (*CXCL*)*9*, and *CXCL10* (Figure 5a,b). A heatmap was prepared for comparative visualization of the chemokines and their corresponding receptors with enhanced gene expression (Figure 5c). The chemokine genes *CXCL9* and *CXCL10* were not included because of negligible expression of their receptor *CXCR3*. Similarly, because the chemokine receptor genes *CCR3*, *CCR8*, G‐protein‐coupled estrogen receptor (*GPER1*), and phosphatidylinositol transfer protein membrane‐associated 3 (*PITPNM3*) showed almost no expression, they were omitted from the heatmap for better visualization.

Upregulated expression of *CCR4* for *CCL5*, *CCR6* for *CCL18*, and *CCR7* for *CCL19* was observed in lymph nodes, where lymphocytes were replaced by lymphoma cells (Figure 5c). The expression of *CCL5*, *CCL18*, and *CCL19* was further upregulated in WDL with prominent lymphoma cell infiltration compared with that with scant lymphoma cell infiltration.

After WDL resection, the patient presented with impaired consciousness and convulsions and was treated with levetiracetam. Infection and rhabdomyolysis were suspected; however, the symptoms resolved. In 2023, a biopsy of the subcutaneous mass in the left axilla revealed lymphoma cell infiltration, and the patient began receiving tirabrutinib for LPL. Although a temporary discontinuation was necessary due to pneumonia, he has been continuing treatment for a year with a minor response with follow‐up CT scan every 4–6 months. WDL is followed up with magnetic resonance imaging every 6 months, but there has been no recurrence for 2 years after resection.

## 3 Discussion

This report presents a case of a tumor with mixed WDL and LPL. A similar case of simultaneous occurrence of gastric diffuse large B‐cell lymphoma and retroperitoneal WDL in a patient with chronic hepatitis B, as well as a case of inflammatory WDL in the retroperitoneum following DLBCL treatment, has been reported [8, 9]. To our knowledge, this is the first case report of LPL infiltration and growth in a patient with WDL.

Although WDL often undergoes dedifferentiation and intermingles with cells exhibiting differentiation other than the adipocytic lineage, there are no reports of WDL differentiating into lymphomas. B‐cell lymphoma may transform into histiocytic sarcoma [10], interdigitating dendritic sarcoma [11], or Langerhans cell sarcoma [12]. In a case of CLL/SLL combined with LCS, it was reported that LCS arose from CLL/SLL, as both cases showed loss of 6q23. In this case, identical genetic mutations were not found in the WDL and LPL regions, which did not support transformation. Only two cases of lymphoma differentiating beyond the hematopoietic system have been reported, in which mantle cell lymphoma was transformed into a sarcoma with neuromuscular differentiation [13]. However, the transformation of lymphomas into adipose tumors has not been reported.

Autoimmune disorders and chronic bacterial infections contribute to the development of low‐grade B‐cell lymphomas of the mucosa‐associated lymphoid tissue (MALT) type [14]. Here, the patient tested positive for HBsAg, but hepatitis or liver dysfunction was not detected via blood tests. A fibrous capsule was observed around the area of lymphomatous growth. We presumed it was an enlarged lymph node with lymphoma cell infiltration. However, owing to its large size (80 mm in diameter), it appeared to be integrated with the WDL in gross view. While the lymph node specimens did not contain intervening CDK4‐positive cells in the background of lymphoma, the CDK4‐positive cells intervened in the background of lymphoma growth in the thigh tumor, suggesting that the lymphoma cells grew within the WDL.

Two completely unrelated tumors may merge sporadically. However, the tumors observed in our patient are very rare, and they appeared to have grown in the same area as if they had an affinity for the same region. Lymphoma cells infiltrate the bone marrow and lymph nodes and spread throughout the body. The highest SUVmax in the thigh tumor area suggested that lymphoma cells were aggressively growing within the WDL region, forming the largest mass. Therefore, we suspected that these two tumors may be inter‐related.

Chemokines (chemotactic cytokines) and their receptors play important roles in establishing normal and neoplastic lymphatic structures [15, 16, 17, 18, 19]. The transcriptome analysis using RNA sequencing revealed that the expression of chemotactic factors, including *CCL5*, *CCL13*, *CCL18*, *CCL19*, *CXCL9*, and *CXCL10*, was significantly upregulated in WDL compared to that in non‐neoplastic fat tissues [20, 21, 22]. Hence, chemotactic attraction may play a role in lymphoma cell infiltration in WDL. The expression of chemokine receptors, *CCR4* for *CCL5*, CCR6 for *CCL18*, and CCR7 for *CCL19*, was upregulated in lymph nodes with conspicuous lymphoma infiltration, likely representing their expression in lymphoma cells. The result raised the possibility that the chemokine–chemokine receptor axes may have been implicated in the infiltration of lymphoma cells into the WDL tissue in this case. Here, *CCL18* was the most abundantly expressed chemokine gene in WDL when compared to the genes in non‐neoplastic fat tissues. Although CCL18 expression was negligible in the lymph node, it was further upregulated in WDL with prominent lymphoma cell infiltration. Therefore, the *CCL18–CCR6* chemokine–receptor interaction might be involved in lymphoma cell infiltration into WDL.

In thymoma, lymphoma develops within a tumor [22]. Monoclonal MALT lymphomas have been identified within MNT [23]. The neoplastic epithelium of MNT expresses high levels of lymphocyte chemotactic or survival factors, including *CCL18*. Aberrant chemokine expression in MNT suggests that they may promote the emergence of monoclonal B‐cells and might disrupt B‐cell homeostasis, promoting the development of mediastinal lymphoma in a chronic inflammatory state [22]. Therefore, in this case, the WDL cells, instead of the neoplastic epithelium MNT cells, abnormally expressed chemokines, including *CCL18*. This may be implicated in the infiltration of lymphoma cells and lymphoma development. Although *CCR3*, *CCR6*, *CCR8*, *PITPNM3*, and *GPER1* encode receptors of *CCL18*, only *CCR6* was expressed in the lymphoma cells. CCL18 exerts multiple functions through its receptors; however, the biological function of *CCL18–CCR6* is unknown [24].

Some WDLs, diagnosed as inflammatory WDLs, exhibit high lymphocytic infiltration. As it is possible that some cases diagnosed with inflammatory WDL are complicated by lymphoma, the presence of enlarged lymph nodes or evaluation of lymphocyte monoclonality may need to be considered.

For the present case, RNA sequencing indicated that WDL cells expressing chemokines might be involved in the pathogenesis of lymphoma. As immunohistochemical analysis of chemokine receptors in FFPE specimens is difficult, owing to the small amounts of receptor proteins and the lack of appropriate primary antibodies, we did not confirm the presence of predicted chemokine–chemokine receptor interactions morphologically. More cases with precise analyses are required to elucidate the mechanism underlying the involvement of WDL in lymphoma tumorigenesis.

## Author Contributions

K.W. (first author and corresponding author) reviewed the data and literature and revised the manuscript accordingly. R.K., S.S., K.K., and S.I. are experts in genetic medicine and provided resources and expertise for genetic interpretation and treatment strategies. Y.N., H.T., and R.S. are clinicians involved in case treatment and management and provided practical clinical findings to the first author. N.N., M.T., C.H., E.Y., Y.O., and T.Y. advised K.W. on the pathological findings of the case and partially revised the manuscript. Y.M. provided the resources and expertise for genetic analysis and revised the manuscript.

## Ethics Statement

This study was conducted in accordance with the tenets of the Declaration of Helsinki. All data used in this study were obtained during the course of medical care required for the patient, and a comprehensive consent form was filled out by the patient. This study was approved by the Research Ethics Review Committee of Kanagawa Cancer Center (approval no. 2021 epidemiology‐101).

## Consent

Written informed consent was obtained from the patient for the publication of the case.

## Conflicts of Interest

The authors declare no conflicts of interest.

## Data Availability

The data that support the findings of this study are available on request from the corresponding author. The data are not publicly available due to privacy or ethical restrictions.
